# The Good, the Bad and the Plenty: Interactive Effects of Food Quality and Quantity on the Growth of Different *Daphnia* Species

**DOI:** 10.1371/journal.pone.0042966

**Published:** 2012-09-25

**Authors:** Tibor Bukovinszky, Antonie M. Verschoor, Nico R. Helmsing, T. Martijn Bezemer, Elisabeth S. Bakker, Matthijs Vos, Lisette N. de Senerpont Domis

**Affiliations:** 1 Department of Aquatic Ecology, Netherlands Institute of Ecology (NIOO-KNAW), Wageningen, The Netherlands; 2 Department of Terrestrial Ecology, Netherlands Institute of Ecology (NIOO-KNAW), Wageningen, The Netherlands; 3 Ingrepro b.v., Borculo, The Netherlands; 4 Wetsus, Centre of Excellence for Sustainable Water Technology, Leeuwarden, The Netherlands; 5 Department of Ecology and Ecosystem Modeling, University of Potsdam, Germany; University of California, Berkeley, United States of America

## Abstract

Effects of food quality and quantity on consumers are neither independent nor interchangeable. Although consumer growth and reproduction show strong variation in relation to both food quality and quantity, the effects of food quality or food quantity have usually been studied in isolation. In two experiments, we studied the growth and reproduction in three filter-feeding freshwater zooplankton species, i.e. *Daphnia galeata x hyalina*, *D. pulicaria* and *D. magna*, on their algal food (*Scenedesmus obliquus*), varying in carbon to phosphorus (C∶P) ratios and quantities (concentrations). In the first experiment, we found a strong positive effect of the phosphorus content of food on growth of *Daphnia*, both in their early and late juvenile development. Variation in the relationship between the P-content of animals and their growth rate reflected interspecific differences in nutrient requirements. Although growth rates typically decreased as development neared maturation, this did not affect these species-specific couplings between growth rate and *Daphnia* P-content. In the second experiment, we examined the effects of food quality on *Daphnia* growth at different levels of food quantity. With the same decrease in P-content of food, species with higher estimated P-content at zero growth showed a larger increase in threshold food concentrations (i.e. food concentration sufficient to meet metabolic requirements but not growth). These results suggest that physiological processes such as maintenance and growth may in combination explain effects of food quality and quantity on consumers. Our study shows that differences in response to variation in food quality and quantity exist between species. As a consequence, species-specific effects of food quality on consumer growth will also determine how species deal with varying food levels, which has implications for resource-consumer interactions.

## Introduction

Growth and reproduction of consumers are not only determined by the quantity of the available food, but also by its quality. While food quantity is rather straightforward to describe and study, the concept of food quality is more complex as it may comprise aspects as diverse as palatability, amounts of organic molecules (e.g., [essential] fatty or amino acids), trace elements, toxins or (macro) nutrient content. Ecological stoichiometry [1] is the domain of science that uses the ratio between carbon (C, energy) and macronutrients such as nitrogen (N) and phosphorus (P) to describe food quality for heterotrophs. Typically, the carbon: nutrient ratio of autotrophs is higher, and the variation larger than that of herbivores [2]. Consequently, herbivores often face nutrient limitation. When subjected to severe phosphorus deficiency (i.e., high C∶P ratios), consumer growth may cease completely, even when food quantity is not limiting [3], potentially leading to strong population declines that may even cascade to higher trophic levels [4]. At moderate levels of nutrient deficiency, consumers may partially compensate for the deleterious effects of poor food quality on growth by increasing feeding rates [5]–[8]. Although these observations suggest that food quality and food quantity are not interchangeable dimensions of food, yet strongly interrelated, most studies tend to examine effects of food quality or food quantity in isolation.

Over the past decades, the freshwater Cladoceran genus *Daphnia* (‘water flea’) has become a model system for studying the effects of nutrient limitation on life-history traits of herbivores [1]. Considerable effort has been paid to explore the role of P-limitation of food in growth and reproduction of *Daphnia*, and studies have found large variation in responses across and within different *Daphnia* species [9]–[14]. Besides growth and reproduction (i.e. production processes), resources are also required for somatic maintenance, which is the collection of processes such as turnover of structural mass (e.g. proteins), respiration, and physical exercise related to foraging (e.g. filtering, processing food) [15], [16].

To understand the growth responses of herbivores to nutrient-deficient food, more insight is needed on how they allocate the limiting nutrients during growth and development. The growth rate hypothesis (GRH) predicts a positive relationship between consumer P-content and growth rates. This relationship is positive because higher growth rates require higher rates of allocation of phosphorus into ribosomal RNA in order to support fast protein synthesis [17]. The slope of this relationship relates to the efficiency at which P is allocated to growth, whereas the intercept is the P-content of animals at ranges of P-limitation where growth ceases. There is variation across organisms both in the intercept and the slope [17]. A study by Kyle et al. [18] showed that the intercept of the relationship between consumer P-content and growth rate differed between *Daphnia* species. They proposed that this variation in intercepts might relate to differences in the nutrient requirements for maintenance [18]. As the phosphorus content of food further increases, growth rates tend to show a unimodal response to P-enrichment, which may relate to the costs of excreting excess nutrients [19]. Studies comparing growth responses of herbivores in response to P-content of food often focus on ranges of extreme nutrient limitation. However, relatively few studies have considered situations where nutrient limitation also include or surpass optimal ranges for growth.

The impact of food quality on herbivore growth depends on the physiological processes to which nutrients are allocated during development. Consumers that are in their early juvenile stage require nutrients predominantly to support somatic growth, while an increasing amount of nutrients is being allocated into reproductive tissue as development nears maturation [15], [20]. Subsequently, growth rates are expected to decrease during juvenile development as allocation of resources to growth is gradually traded off against allocation to reproduction. During development, *Daphnia* displays interspecific variation in resource allocation to growth and reproduction with consequences for body nutrient content [21]–[23]. Thus, interspecific differences in responses to food quality may depend on ontogenetic differences in the coupling of growth rates and body P-content, but this aspect remains largely unexplored.

The different requirements of energy and nutrients for growth and maintenance relate to the observed pattern that the effect of dietary P–limitation on consumer growth depends on the quantity of available food [24]. Maintenance is expected to largely depend on carbon, but it may also require nutrients such as phosphorus [18], [25]. One way to examine the possible role of carbon and phosphorus in maintenance is to compare the effects of different phosphorus levels of food (C∶P ratios) on the minimal residual food concentrations. The latter are called the food threshold concentrations (*C_0_*), where food levels support maintenance only. If maintenance requires only carbon (energy), the negative effect of P-limited food on growth should decrease and disappear as food concentrations decline to *C_0_* [5]. In contrast to this expectation, Boersma & Kreutzer [7] reported an increase of *C_0_* of *Daphnia magna* when developing on P-deficient food. This increase was attributed to the possibly higher amount of carbon (energy) required to process P-limited food. Alternatively, it may be possible that P-requirements for maintenance result in an increase of *C_0_* under P-limitation. A testable hypothesis that follows from this result is that species with higher estimated P-content at zero growth should respond to phosphorus–limitation of food with a stronger increase in their threshold food concentrations (*C_0_*). Both intake rates and metabolic costs depend on body size within the genus *Daphnia* [26], [27]. Larger *Daphnia* filter more efficiently and thus should have lower *C_0_* than smaller species [28]. Also, larger species are expected to have lower relative metabolic costs (per unit of body mass) than smaller species [27].

Using different *Daphnia* species fed with the green alga *Scenedesmus obliquus* (Turpin) Kützing as a study system, we carried out two experiments in order to study i) the relationship between growth rate and body-P content of different herbivore species, and ii) the changes in threshold food concentrations (*C_0_*) of different herbivore species under P-limitation of food. In these experiments three *Daphnia* species were used, the small *D. galeata x hyalina*, the intermediately-sized *D. pulicaria* and the large *D. magna*. We expected an increase of *C_0_* when animals were provided with P-poor food. Furthermore, we expected that a species with a higher estimated P-content, should also respond to phosphorus–limitation of food with a stronger increase in its *C_0_*. We hypothesized that the nutrient requirements for maintenance and growth are different between *Daphnia* species, which will be reflected in their growth responses and *C_0_* on P-poor and P-rich food.

## Methods

### Culturing algal food

Single-celled *Scenedesmus obliquus* (strain originating from the Max Planck Institute of Limnology, Plön, Germany) was used as food for *Daphnia* in all experiments. *S. obliquus* was cultured on P-deficient WC medium (10 µmol P L^−1^) [29]. To maintain constant food quality (C∶N∶P), algae were cultured in continuous 1 L volume chemostats (light intensity 120 μmol [PAR] m^−2^ s^−1^, 0.039% CO_2_) and at equilibrium conditions, at a dilution rate of ∼0.25 d^−1^, resulting in algae with a molar C∶P ratio of ∼750.

Studies of phosphorus limitation often focus on ranges along which zooplankton cannot mature and experience completely inhibited reproduction, whereas C∶P ratios in lakes often vary much less [30]. Therefore we selected C∶P ratios that allowed *Daphnia* to reach adulthood and that did not completely inhibit growth or reproduction. In addition, these more realistic C∶P ratios will allow for a better translation of a proof of principle to *in situ* conditions. Most studies suggest a threshold elemental ratio between 300 and 200 for *Daphnia* [31]. To manipulate nutrient content of algal food, batches of P-deficient S. obliquus were enriched by adding different concentrations of K2HPO4. After adding phosphorus to batches of algae collected from the effluent of the chemostat, algae were kept in the dark for at least two hours to ensure uniform conditions for taking up phosphorus. Subsequently, algae were added to the zooplankton culture medium and algae with C∶P ratios of approximately 400 (P- poor), 200 (P- sufficient) and 50 (P- rich) were obtained. Pulsing P-deficient *S. obliquus* ensured that the effects studied on *Daphnia* here were caused by P-enrichment alone (Table 1). Moreover, this method ensured that no other effects, such as allocation of carbon to different molecules at low and high P-levels could have confounded our experimental treatments [10]. In the second experiment, C∶P 400 and C∶P 80 foods were used.

**Table 1:** Mean (standard error) food quality expressed in molar C∶N and C∶P ratios for stock cultures (chemostat), P-poor, P-sufficient and P- rich treatments used in the two experiments.

	Experiment 1		Experiment 2	
	*C*∶*P ratio*	*C*∶*N ratio*	*C*∶*P ratio*	*C*∶*N ratio*
**Chemostat**	770.9 (32.9)	13.9 (0.1)	729 (21.6)	12.2 (0.3)
**P-poor**	390.8 (8.5)	13.5 (0.1)	418.1 (8.8)	11.7 (0.4)
**P-sufficient**	195.4 (5.4)	13.4 (0.1)	-	-
**P-rich**	48.8 (1.7)	13.3 (0.1)	81.1 (3.1)	11.7 (0.4)

### Zooplankton

Clones of *Daphnia magna* Straus, *D. pulicaria* Forbes and a *D. galeata x hyalina* hybrid were maintained at the culture facilities of the Department of Aquatic Ecology of the Netherlands Institute of Ecology (NIOO-KNAW). *D. magna* is the largest of the three species, with ca. 7.5 μg dry weight (DW) as a neonate, followed by *D. pulicaria* (∼6 μg DW) and *D. galeata x hyalina* (∼1.9 μg DW). These clones were maintained in 100 mL tubes with membrane (0.45 µm) filtered mesotrophic lake water (Lake Maarsseveen), and were fed excess (0.5 mg C L^−1^) phosphorus-sufficient (∼180 C∶P) *S. obliquus*. In order to eliminate age-related developmental effects, neonates of the third or later broods born within 6 to 8 hours from gravid females were used as experimental animals. Experimental animals were pooled and distributed randomly into flow-through vessels. At start-up, a random subsample was taken to measure neonate DW, C, N and P-content of different species/clones.

### Experimental set-up

Throughout the experiments, animals were kept in 140 mL flow-through microcosms made of poly(methyl methacrylate) [32]. Flow-through vessels were suspended in thermostatically controlled water-baths at 20±1°C, and were kept under dim light and at 16∶8 h (Light: Dark) regime. Every day, algal food was added to vessels containing zooplankton medium, according to the experimental treatments (Table 1). The zooplankton medium was a mixture of 80% artificial *Daphnia* medium ADaM [33] and 20% of 0.45 µm membrane-filtered water from an open reservoir of phosphate-depleted drinking water (Utrechtse Waterleidingplas). We selected this mixture as preliminary work revealed that pure ADaM negatively influenced the survival of *Daphnia* juveniles. The mixture of natural P-free lake water and pure ADaM alleviated this problem without affecting phosphorus levels in the medium. Throughout the experiments, the P-content of the experimental medium was below the detection limit (<1 μg P L^−1^) of the segmented flow autoanalyzer (QuAAtro, Seal analytical Inc., Beun de Ronde, Abcoude, The Netherlands). The algal suspension was kept in the dark while being fed to the flow-through vessels by two multi-channel peristaltic pumps at a rate of 20 mL per hour. At the bottom of each microcosm the outlet was covered with a 60 μm mesh to allow medium and algae to pass through, but not the zooplankton. The measured relationships between particulate carbon content (for description of chemical analyses see below), photospectrometer measurements of absorbance (optical density at 750 nm) and cell density (using a CASY electronic particle counter, Schärfe System Gmbh, Reutlingen, Germany) were used to standardize the carbon concentration in the experimental zooplankton medium.

### Experiment 1

Phosphorus-poor (C∶P 400), P-sufficient (C∶P 200) and P-rich (C∶P 50) algal food were fed to animals at concentrations (0.5 mg C L^−1^) that were more than sufficient for all three *Daphnia* species [10], [34]. For each treatment, neonates were distributed in five replicated (140 ml) vessels, where the number of individuals per vessel was 12 for *D. magna*, 16 for *D. pulicaria* and 20 for *D. galeata x hyalina* to standardise initial biomass among different species treatments. After four days, half of the animals were harvested from each vessel and taken for subsequent chemical (C, N, P) analysis. When animals in a vessel reached adulthood (i.e. first clutch of eggs was released in the brood chamber), all remaining animals were harvested, the number of eggs per female was counted and dry mass was measured. Subsequently, samples were taken for C∶N∶P measurements.

### Experiment 2

Phosphorus-poor algal food (C∶P 400) was compared to P-rich (C∶P 80) algae. Food concentrations were standardized at four treatment levels, 0.08, 0.14, 0.2 and 0.3 mg C L^−1^. For each treatment level, neonates were distributed in three to four replicated (140 ml) vessels, where the number of individuals per vessel was 6 for *D. magna*, 8 for *D. pulicaria* and 14 for *D. galeata x hyalina*. Due to limited numbers of experimental animals, for two C-concentration treatments (0.08 and 0.2 mg C L^−1^) of *D. magna*, only two replicated vessels were available. After four days, animals were harvested for C-, N-, P measurements.

### Chemical analysis

For the chemical analysis of algal samples, the suspension was filtered through pre-combusted (at 500°C) glass fiber (GF/F, Whatman) filters and dried at 60°C overnight. For measurement of P-content of seston retentate and *Daphnia*, samples were first incinerated for two hours at 500°C, followed by 2% potassium persulfate (K_2_S_2_O_8_) digestion. Phosphorus content was determined using a QuAAtro segmented flow autoanalyzer. C- and N-content in seston retentate and *Daphnia* were determined using a FLASH 2000 organic elemental analyzer (Brechbueler Inc., Interscience B.V., Breda, The Netherlands).

### Data analysis

Individual specific growth rates GR (day^−1^) were expressed for each vessel as:
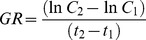
where *C* is body mass in carbon (μg individual^−1^) at successive times t_1_ and t_2_. Individual specific growth rate is a robust alternative to measuring fitness and intrinsic population growth rate as it encompasses most of the variation due to age or maternal effects [35]. In the first experiment, where age of development was included in the analysis, carbon measurements were not available for juveniles of all species. Therefore GR was calculated based on dry weight. The comparison of growth rates derived from dry weight and *Daphnia* carbon content confirmed that this did not affect the interpretation of the results.

In the first experiment, the specific growth rates of the different *Daphnia* species and of different age (i.e. juveniles and when adulthood was reached) were compared using General Linear Models (GLM). First, only the fixed effects (*Daphnia* species, Age, Food quality) were included in the model. Subsequently, the effects of body P-content and N∶P ratios on growth rates were analysed using analysis of covariances (ANCOVA). Visual inspection of the slopes between the covariates (P – content or N∶P ratios) and growth rates suggested a second-order polynomial relationship, which was tested by including the covariates as a quadratic term in the models. The extrapolated P-contents of *Daphnia* species at zero growth rates were found by finding the roots of the fitted polynomial relationship. In order to test for differences between slopes in the relationship between P – content and growth rate across treatment groups, we linearized the data by a reciprocal (1/x) transformation of the covariate before performing the ANCOVA. Both models, where the covariate (P- content) was either included as a quadratic term or it was linearized, gave qualitatively the same outputs in terms of main effects and interactions. The relationship between growth rates and clutch size was analysed by linear ANCOVA.

In the second experiment, growth rates were compared by including food concentrations (as ln (mg carbon L^−1^ medium)) as a covariate in the ANCOVA, with species and C∶P ratios of food as fixed factors. Inferences were based on the full model (all interactions included). The fit of the models used for final inferences was based on testing for normality of the residuals, using a Kolmogorov-Smirnov test.

To determine the threshold food concentrations for zero growth, the relationship between carbon concentrations of food and specific growth rates (GR) of *Daphnia* on different C∶P ratios of algal food were analyzed. A non-linear regression model [36]; was fitted to the consumer growth data [37], [38], with a threshold for zero growth:
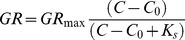
where *GR_max_* (day^−1^) is the maximum specific growth rate, *C* is the food concentration (mg C L^−1^), *C_0_* is the threshold *C* concentration that supports zero specific growth rates, and *K_s_* is the half-saturation constant (mg C L^−1^). All analyses were performed using SAS 9.1 (SAS Institute Inc., Cary, NC, USA).

## Results

### Growth rates, body stoichiometry and clutch size

**Figure 1:**
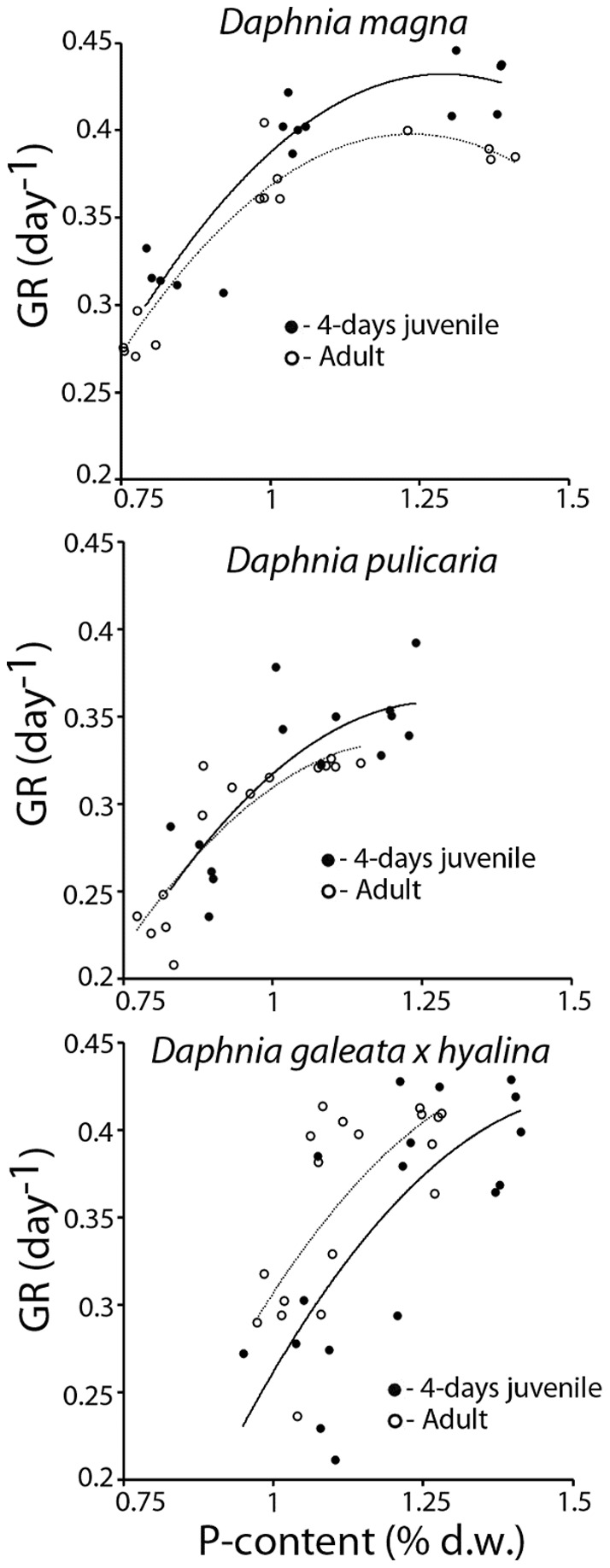
Mass-specific P-content in relation to specific growth rates of *D. magna*, *D. pulicaria* and *D. galeata x hyalina*, after four days (closed symbols) and at maturation (open symbols). Lines were fitted using ANCOVA with quadratic term for P-content.

In the first experiment, the specific growth rates of the three *Daphnia* species showed considerable variation (GLM, Species: F_2,90_ = 10.53, P<0.001), with similar average values for *D. magna* (0.366 day^−1^) and *D. galeata x hyalina* (0.355 day^−1^) and a significantly lower growth rate for *D. pulicaria* (0.304 day^−1^). In general, juvenile animals had higher specific growth rates than maturing animals (GLM, Age: F_1,78_ = 15.67, *P*<0.001), and this difference was observed both for *D. magna* (*P* = 0.003) and for *D. pulicaria* (*P* = 0.005). However, no overall effect of developmental age on growth rates was found for the smaller *D. galeata x hyalina* (*P* = 0.927). P-content had a strong positive effect on growth rates (Fig. 1). Specific growth rates increased stronger with P-content at the lower ranges of the relationship, resulting in a curvilinear relationship for each species. The non-linear effect of P-content on growth rates was reflected by the significant effect of body P-content as a quadratic term in the analysis (F_1, 84_ = 13.95, P<0.001).

The extrapolated P-content at minimum growth rates (intercept with x-axis) decreased with body size, being lowest for the large-sized *D. magna*, intermediate for *D. pulicaria* and high for the small-sized *D. galeata x hyalina* both for maturing animals and juveniles. For maturing animals, the P-contents at zero growth were different between the three species, and the estimated mean values (±95% confidence limits) were for *D. magna* 0.4% (0.36–0.44), for *D. pulicaria* 0.52% (0.57–0.62) and for *D. galeata x hyalina* 0.76% (0.68–0.84). A similar trend for increasing zero growth with decreasing species body size was observed for juvenile animals, although in this case the confidence intervals overlapped. The estimated P-content at zero growth for juvenile *D. magna* was 0.32% (0.25–0.41), for *D. pulicaria* 0.44% (0.29–0.56) and for *D. galeata x hyalina* 0.5% (non. estimable –0.79).

In order to test for differences between slopes of different treatment groups, we linearized the data by reciprocal (1/x) transformation of the covariate. When entering P-content as a covariate in the model, the effect of age of animals on growth rates, as well as the interaction between age and P – content, were not significant (Table 2). This indicated that the couplings between body P-content and growth rate were similar for juvenile and maturing animals (Fig. 1). Our analysis also revealed an interaction between developmental stage and species identity (‘Age’ and ‘Species’; F_2, 84_ = 6.34, P = 0.003, Table 2). This implied that species showed a different pattern of response between developmental stages, which was indicated by the different reaction norms shown in Fig. 1. Furthermore, our linearized model showed that the slopes of the relationship between growth rate and body P – content were different across *Daphnia* species (P – content * Species, F_2, 84_ = 8.04, P<0.001): the slope for *D. galeata x hyalina* was steeper than either that of *D. pulicaria* (P = 0.004) or *D. magna* (P<0.001), whereas similar slopes were observed for *D. pulicaria* and *D. magna* (P = 0.394).

**Table 2:** Results of ANCOVA for the effects of *Daphnia* body P-content and age (4-day-old juveniles, mature adults) on specific growth rates (GR) of *D. magna, D. pulicaria* and *D. galeata x hyalina*.

Source	df	SS (type 3)	F-value	P-value
**P-content**	1	0.166	136.22	<0.001
**Species**	2	0.014	5.80	0.004
**Age**	1	0.0	0.22	0.641
**Species x Age**	2	0.015	6.34	0.003
**P-cont x Species**	2	0.019	8.04	<0.001
**P-cont x Age**	1	0.0	0.28	0.599
**Error**	85	0.103		

The explanatory variable “P – content” was linearized before analysis.

**Figure 2:**
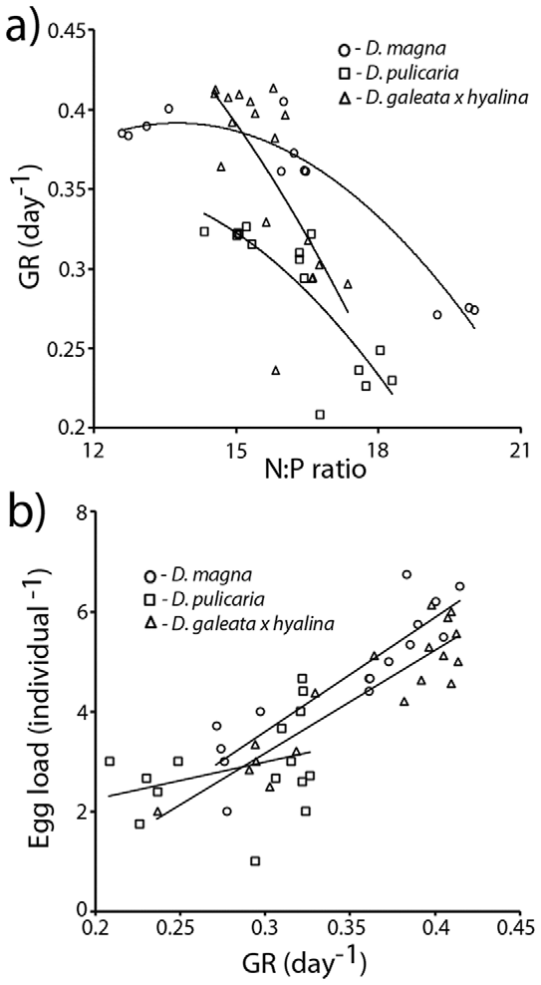
Relationships between A) specific growth rates and N∶P ratios, and B) specific growth rate and clutch size for *Daphnia magna* (circles), *D. pulicaria* (squares) and *D. galeata x hyalina* (triangles). Lines were fitted using ANCOVA.

The differences in growth rates and P-content of adult animals were also reflected in changes in their body N∶P ratios. There was a negative correlation between body N∶P ratios of adults and specific growth rates (Fig. 2A) and this relationship differed significantly between *Daphnia* species (N∶P ratio x Species interaction, F_2,38_ = 6.85, *P* = 0.003). The relationship appeared to be the most negative for *D. galeata x hyalina* (Fig. 2A). Specific growth rates positively correlated with clutch size (F_1,40_ = 51.32, *P*<0.001, Fig. 2B), and the slope of this relationship varied across species (F_2,40_ = 3.28, *P* = 0.048). The slope was significantly lower for *D. pulicaria* than for either of the two other species (*D. pulicaria vs D. magna*, F_1,40_ = 4.9, *P* = 0.033, *D. pulicaria vs D. galeata x hyalina*, F_1,40_ = 5.34, *P* = 0.026), while the slopes of *D. magna* and *D. galeata x hyalina* were similar (F_1,40_ = 0.08, *P* = 0.775).

### Phosphorus limitation, food concentration and growth

**Figure 3:**
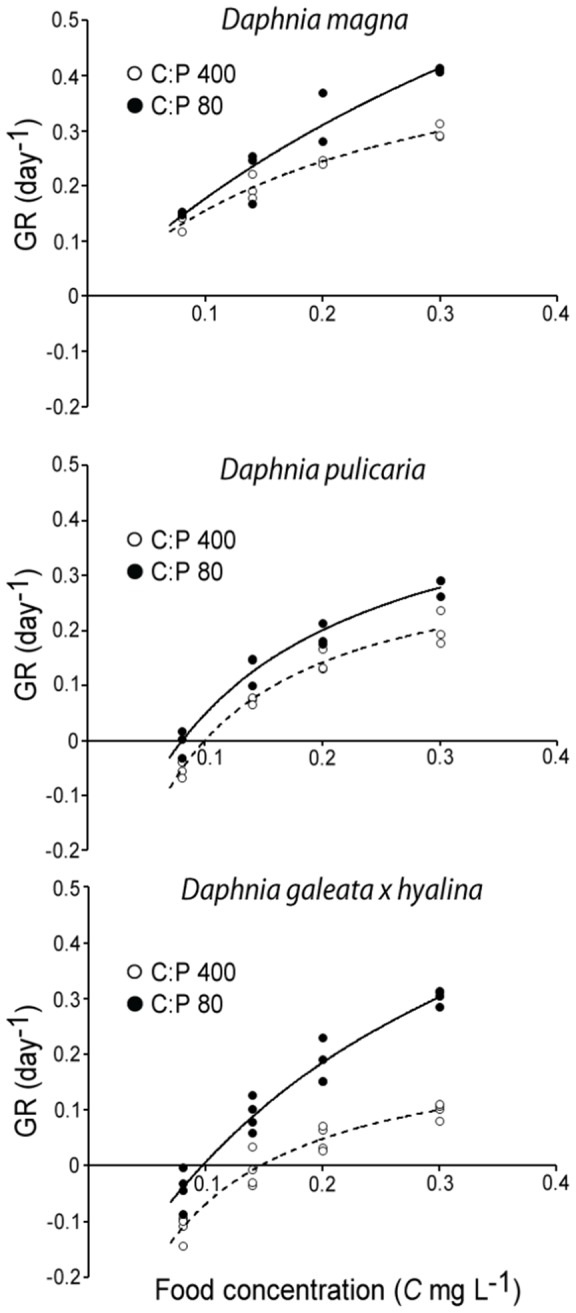
Specific growth rates (GR) of 4-day-old *D. magna*, *D. pulicaria* and *D. galeata x hyalina* cultured on P-poor (C∶P 400; open symbols) and P-rich (C∶P 80, closed symbols) algal food at four food concentrations (0.08, 0.14, 0.2 and 0.3 mg C L^−1^). Lines were fitted using the Monod model.

In the second experiment, average specific growth rates increased with body size across the three species (*D. galeata x hyalina*: 0.07 day^−1^; *D. pulicaria*: 0.12 day^−1^; *D. magna*: 0.25 day^−1^). Food concentrations (F_1,65_ = 1010.8, *P*<0.001; Fig. 3) and P-content (F_2,65_ = 76.00, *P*<0.001) both had strong positive effects on specific growth rates. The differences between growth rates of different *Daphnia* species were pronounced on P-poor food at all concentrations, while the differences between *Daphnia* species became less clear at high food concentrations, especially when fed P-rich food. These patterns were reflected in the species-dependent effects of food concentrations on *Daphnia* growth (Food concentration*Species, F_2,65_ = 4.47, *P* = 0.015), and in the species-dependent effects of P-content of food on growth rates (Phosphorus *Species, F_2,65_ = 8.31, *P*<0.001).

**Figure 4:**
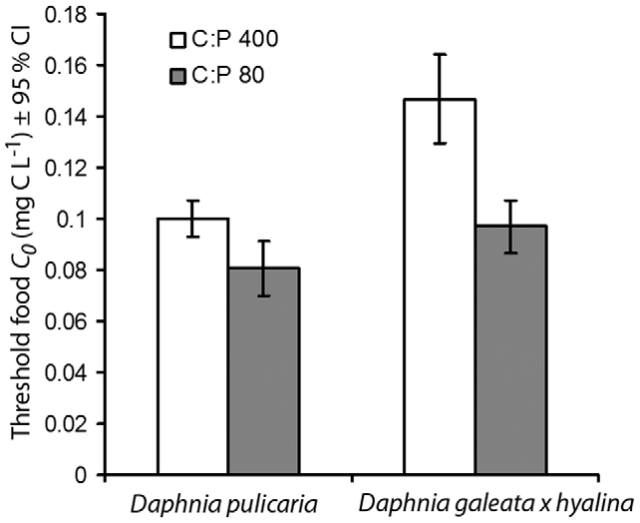
Threshold food concentrations (*C_0_*±95% CI) supporting zero specific growth rates of 4-day-old *D. pulicaria* and *D. galeata x hyalina* cultured on P-poor (C∶P 400, open bars) and P-rich (C∶P 80; filled bars) algal food. *C_0_* values are parameter estimates from the Monod model.

Threshold food concentrations could be estimated with confidence for *D. galeata x hyalina* and *D. pulicaria*, but not for *D. magna*. Threshold concentrations supporting zero specific growth rates (*C_0_*) were lower for the larger *D. pulicaria* than the smallest species, *D. galeata x hyalina*. The effect of phosphorus enrichment of food diminished as food concentrations decreased (Food concentration*Phosphorus, F_1,65_ = 27.9, *P*<0.001; Fig. 3), but did not completely disappear as effects of P-content of food were still present at *C_0_* (Fig. 4). Threshold food concentrations were higher on P-poor than on P-enriched food for both *Daphnia* species and the confidence intervals of *C_0_* on P-poor and P-rich food did not overlap. Compared to the *C_0_* observed on P-poor food, P-enrichment reduced *C_0_* for *D. galeata x hyalina* by 34%, whereas this reduction was 19% for *D. pulicaria* (Fig. 4).

## Discussion

The aim of our study was to examine how food quality (i.e. stoichiometric constraints) and food quantity together influenced growth of *Daphnia* species. In the first experiment, we found a generally positive relationship between specific growth rates and body P-content, which was in line with the prediction of the growth rate hypothesis (GRH) that faster growth requires disproportionately more P than slower growth [1], [17]. Our study showed interspecific variation in the coupling between the growth rate and P-content of consumers. Variation in slopes reflects differences in nutrient requirements for growth, whereas variation in P – content of animals at minimum growth rates reflects differences in nutrient requirements for maintenance. The relationship between P – content and growth rate was steeper for *D. galeata x hyalina* than for the other two species, whereas slopes of *D. magna* and *D. pulicaria* were similar. The estimated x-intercepts at zero growth suggested the lowest body P – content for the largest *Daphnia magna*, intermediate for *D. pulicaria* and highest for *Daphia galeata x hyalina*. This variation in body – P content at minimum growth rates suggested that the three species may have different phosphorus requirements for maintenance.

An earlier study by Kyle et al. [18] showed species-specific differences in the intercepts, relating these differences to variation in P – requirements for maintenance. Kyle et al. [18], however, did not observe differences in the slopes of the relationship between body – P content and growth rates. The differences between our study and that of Kyle et al. [18] may reflect methodological differences, which in turn yield interesting insights into the mechanisms behind phosphorus-limited growth. The two studies used different methods to standardise quality and levels of food. We pulsed algae with phosphorus in order to prevent confounding effects of other food quality parameters that covary with phosphorus over longer growth periods, such as morphology [39] or fatty acid composition [3]. The two studies also used different clones of the same species, and interclonal variation in rRNA polymorphism (and thus phosphorus requirements) is known to mediate the effects of P-limitation on growth rates [40]–[42]. The variation in slopes among *Daphnia* species observed in our study was also likely related to the non-linear growth response of *Daphnia* to P-enrichment of food. At maximum growth, nutrient demands are optimally met, and further nutrient increases in the food supply will result in a monotonic decrease in growth rates. This may be explained by metabolic costs associated with excretion of the excess of nutrients in order to maintain homeostasis [43]. As a result, herbivore growth responses over a gradient of phosphorous contents in food are expected to show a unimodal response pattern (“stoichiometric knife-edge effect” or “too much of a good thing”) [19]. Levels of P-limitation applied in other studies [18] are typically more severe (C∶P around 1000 for low quality treatments, and around 100 for high quality treatments) and restrict growth responses to the range where obvious P-limitation occurs. In contrast, C∶P ratios in our study ranged from moderate limitation (400) to sufficient (200) or perhaps even surplus (50), where growth may no longer be limited by phosphorus. In our study, growth rates gradually levelled off at higher P-content, which was especially pronounced in the case of *D. magna*. Also in the second experiment, such a “knife-edge effect” may have decreased the observed effects of food quality on C_0_ of *Daphnia*. Thus interspecific variation in the growth response of herbivores to high P – contents of food may not only be important for phylogenetically unrelated taxa [19], but also for closely related species like Cladocerans [10]–[12]. Future studies may consider scenarios where growth rates of herbivores are evaluated within a broad range of nutrient concentrations. This will not only shed light at ecophysiological processes under conditions where obvious nutrient limitation occurs, but also under conditions where nutrient excess inhibits growth, which may be relevant for ecosystems under eutrophication.

We found a significant interaction between effects of food quantity and food quality. As food concentrations decreased, the effects of nutrient limitation weakened. These results are in agreement with the model of Sterner & Robinson [5]. This model also suggests that when food concentrations decrease, the importance of energetic costs will increase and the importance of P-limitation will decrease. In contrast to this model, but in agreement with the findings of Boersma & Kreutzer [7], we found that decreasing the P-content of food increased threshold food concentrations (*C_0_*). In response to P-limitation of food, *C_0_* increased more for *D. galeata x hyalina* than for *D. pulicaria*. *Daphnia galeata x hyalina* was also estimated to be richer in phosphorus at the point where growth halted due to phosphorus starvation. These results suggest that a higher nutritional requirement for maintenance should lead to a larger increase in *C_0_* on P-limited food. Boersma & Kreutzer [7] attributed the increase of *C_0_* of *D. magna* on P-limited food to the higher energetic costs of processing low-quality food. Such an overhead process may refer to the thermic effect of food or specific dynamic action [15]. Filtering rates in *Daphnia* also increase when feeding on food of low P-content. Thus, the energetic costs of physical exercise while filtering are expected to be higher when feeding on P-limited food [16]. Furthermore, the differences in *C_0_* between *Daphnia* species in our study indicate that the effects of P-limitation on *C_0_* may reflect differences in nutritional requirements of maintenance [43]. Maintenance costs may relate to interspecific differences in traits between species such as body size [26]–[28], but it is currently unclear to what extent such traits correlate with energetic costs only or also with nutritional requirements. Our results show that the costs of dealing with changing quality food at low quantities differ across species. Future studies should confirm whether the differences are explained by costs of exercise, costs of maintenance, or by the combination of both.

For the two larger species, *D. magna* and *D. pulicaria*, we found that growth rates decreased as animals grew older, which coincided with a decrease in P-content (an increase in N∶P ratios). Similar results have been found in other studies [20], [44], [45]. The underlying mechanism is that as animals develop and growth rates decelerate, resources are allocated to structural and reproductive tissues differently [15], [20], [21], [23]. Interestingly, for the species *D. pulicaria* and *D. magna*, juveniles and maturing animals exhibited similar relationships (slopes) between growth rate and body P-content. Our finding suggests that although the P-requirements allocated to growth decreases as animals mature, this decrease changes according to species-specific slopes of the efficiency at which phosphorus is allocated to growth.

This study investigated the interdependence of food quality (as phosphorus content) and food quantity in a standardised manner across three closely related herbivore species. Our results show that there are interspecific differences in minimum food levels required to sustain a population (*C_0_*). These differences appear to be dependent on P-content of the food and may be explained by the abilities of different species to allocate phosphorus to growth [43]. Threshold food concentrations for somatic growth also closely correlate with reproductive output and population growth rate in *Daphnia* [34], [35]. Therefore, such variation in life history characteristics across species may influence the outcome of competitive interactions among species under varying food quality [41].
